# The Positive and Negative Effects of Calcium Supplementation on Mortality in Septic ICU Patients Depend on Disease Severity: A Retrospective Study from the MIMIC-III

**DOI:** 10.1155/2022/2520695

**Published:** 2022-06-22

**Authors:** Wencheng He, Lei Huang, Hua Luo, Jingying Chen, Weijia Li, Yiming Zhang, Youzhong An, Weixing Zhang

**Affiliations:** ^1^Department of Intensive Care Unit, Peking University Shenzhen Hospital, Shenzhen Peking University, The Hong Kong University of Science and Technology Medical Center, No. 1120, Lianhua Road, Futian District, Shenzhen 518000, China; ^2^Department of Intensive Care Unit, Peking University Shenzhen Hospital, No. 1120, Lianhua Road, Futian District, Shenzhen 518000, China; ^3^Department of Intensive Care Unit, Peking University People's Hospital, No. 11, Xizhimen South Street, Xicheng District, Beijing 100044, China

## Abstract

**Background:**

Calcium administration in septic patients with hypocalcemia is a controversial issue. The present study preliminarily investigated the effects of calcium supplementation on the length of hospitalization and mortality in septic ICU patients with different severities of hypocalcemia and disease.

**Method:**

A total of 5761 eligible septic patients, including 2689 who received calcium supplementation and 3072 who did not receive calcium supplementation, were extracted from the Medical Information Mart for Intensive Care III (MIMIC-III) database. The cofounding covariates between the calcium supplement and nonsupplement groups were balanced using the propensity score matching model. We compared the length of stay (LOS) in the ICU and hospital with 28-day and hospital mortality and stratified the analysis according to the sequential organ failure assessment (SOFA) score and ionized calcium (iCa) at the first ICU admission in the matched groups.

**Results:**

The results showed that iCa at the first ICU admission was associated with mortality in sepsis patients (HR: 0.421; 95% CI: 0.211∼0.837), but the lowest mortality rate was observed in patients with mild hypocalcemia. A total of 993 paired patients were included in the analysis after propensity score matching. Regardless of the SOFA score or presence of iCa, the LOS in the ICU was higher in the calcium supplement group than in the nonsupplement group. The survival analysis was stratified by the SOFA score and showed that calcium supplementation reduced mortality when the patient's SOFA score was ≥8 (*p*=0.002), and it worsened the outcome when the patient's SOFA score was ≤4 (*p*=0.010). It had no significant effect on patients with SOFA scores ranging from 5 to 7 (*p*=0.911).

**Conclusion:**

Our results showed that mild hypocalcemia may be protective in septic patients, and calcium supplementation may have positive and negative effects on mortality depending on disease severity. The SOFA score may be a valuable clinical index for decisions regarding calcium administration.

## 1. Introduction

Hypocalcemia is a widely recognized biochemical abnormality in critically ill patients [1, 2], and septic patients are at particular risk for hypocalcemia [3, 4]. The etiology of hypocalcemia has been extensively researched in septic patients [5, 6], and many factors, such as decreased parathyroid hormone (PTH) release, tissue calcium accumulation, ionized calcium (iCa) release into ascites fluid, and hypomagnesemia, are involved in causing hypocalcemia during sepsis. However, the major factors that cause hypocalcemia in sepsis are unclear, and more than half of patients in the intensive care unit (ICU) have no identifiable etiology for hypocalcemia.

iCa is a ubiquitous intracellular messenger and coenzyme throughout the body. Without adequate calcium regulation, the body ceases to function properly and many different clinical signs manifest [7]. Hypocalcemia is associated with poor outcomes in septic patients [8–10]. Patients with severe hypocalcemia who fail to recover a normal iCa level during the early stage have significantly higher mortality [2]. Calcium should be replaced to prevent life-threatening complications, such as laryngospasm, tetany, seizures, and cardiac abnormalities [11, 12]. However, the treatment of hypocalcemia in critically ill patients is a controversial issue [13]. We lack evidence-based guidance because no randomized controlled trial has evaluated the effects of calcium administration on prognosis in septic patients. Studies in critically ill patients also yielded conflicting results regarding calcium supplementation. One large retrospective study showed that calcium supplementation improved the 28-day and 90-day survival of ICU patients [14]. However, several clinical observation studies showed that calcium supplementation in critically ill patients provided no benefit or worsened outcomes [15, 16]. Experiments using an animal model of sepsis showed that calcium administration increased mortality [17].

iCa is more reliable and sensitive than total calcium for hypocalcemia evaluation. However, iCa is not a routine measurement in the ICU setting, and some treatments, such as a high dose of albumin or fast transfusion, can easily affect iCa. The identification of a reference index to substitute for iCa or total calcium would be valuable in the decision-making process for clinical calcium administration. Previous studies on the effects of calcium administration on the prognosis of septic patients did not stratify patients according to disease severity. Whether the effects of calcium administration varied in patients with different SOFA scores were not known. Therefore, we performed a retrospective study by extracting the data of sepsis patients from the Medical Information Mart for Intensive Care III (MIMIC-III) database to preliminarily evaluate whether calcium supplementation should be administered during the ICU stay and to identify suitable conditions for calcium supplementation in septic ICU patients. A preprint of the results was previously published [18].

## 2. Methods

### 2.1. Design

The present study preliminarily investigated the effects of calcium supplementation on the prognosis of septic patients in the ICU. Factors including age, sex, iCa, and lactate at the first ICU admission and other complications were examined to determine whether there were associations with hospital mortality in septic patients. The prognostic differences between the calcium-supplemented and nonsupplemented groups were stratified and analyzed according to iCa at the first ICU admission and SOFA scores. The ranges of iCa and SOFA scores that worsened or benefited the outcome were examined using survival analysis.

### 2.2. Database

This study was a single-center, retrospective, observational study. We used the MIMIC-III (latest version 1.4) database. The MIMIC-III is an open, publicly available ICU database that is composed of clinical data and physiological waveforms. The database contains records from 53,423 deidentified ICU patients admitted to the Beth Israel Deaconess Medical Center (BIDMC, Boston, MA, USA) from 2001 to 2012. The database contains records of demographics, intravenous (IV) medications, laboratory results, nursing progress notes, fluid balance, and other clinical variables. Our access to the database was approved after completion of the CITI program course named “Human Research Data or Specimens Only Research” (Record ID: 31532119). The institutional review boards of the Massachusetts Institute of Technology and the BIDMC approved the project, and informed consent was waived due to the purely observational nature of the study. The Ethics Committee of Peking University Shenzhen Hospital approved this study. Data extraction was performed using PostgreSQL (version 4.6).

### 2.3. Cohort Selection

**Figure 1:**
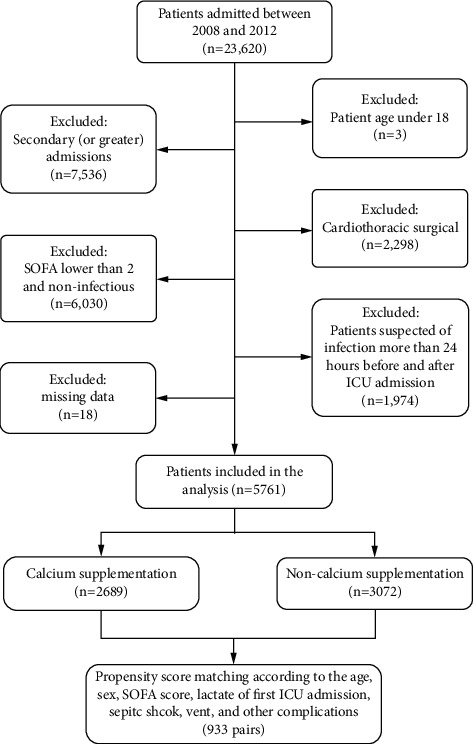
Illustration of the exclusion and inclusion criteria used to select the final cohort of 5,761 patients.

Sepsis patients were identified by the method reported by Alistair et al., which closely adhered to the sepsis-3 definition [19]. Queries were stored on a public repository GitHub (https://github.com/alistairewj/sepsis3-mimic/tree/master/query). The explicit sepsis codes were introduced at the BIDMC in 2004, and the group of admissions between 2008 and 2012 was easily identifiable in the database. Therefore, only ICU admissions from 2008 to 2012 (*n* = 23,620) were enrolled in the present study. The admissions included 3 nonadult patients, 7,536 patients with secondary (or greater) admissions (to avoid repeated measures), 2,298 patients who underwent cardiothoracic surgery (their postoperative physiological derangements did not translate to the same mortality risk as to the other ICU patients), 1,974 patients with suspected infections that lasted longer than 24 hours before and after ICU admission (we focused on the majority of patients who were admitted to the ICU with sepsis to ensure independence between data points because the MIMIC-III only contained ICU data), and 6,030 patients with SOFA scores lower than 2 and were considered noninfectious; 18 patients with missing data were excluded. The final cohort contained 5,761 patients (Figure 1), and 2,689 of these patients received calcium supplementation during their ICU stay.

### 2.4. Statistical Analysis

The normality of the distribution of continuous variables was tested using the Kolmogorov–Smirnov test. Data with a normal distribution are expressed as the mean ± SD and were compared using Student's *t*-test. Otherwise, data are expressed as the median with quartiles and were compared using the Mann–Whitney *U* test. Categorical variables are expressed as percentages and were compared using the chi-squared test or Fisher's exact test as appropriate. Variables between survivors and nonsurvivors were compared using univariate analysis to screen for potential risk factors associated with hospital mortality. Covariates with *p* < 0.1 in the single-factor analysis were included in Cox regression analysis to investigate the significant risk factors associated with hospital mortality. The proportional hazard assumption and the linearity relationship between the covariates and outcome were confirmed by the Schoenfeld residual test, and the linearity relationship between the covariates and outcome was confirmed by the Martale residual test.

To balance the confounding covariates between the calcium-supplemented and nonsupplemented groups, factors that were previously associated with mortality from sepsis, including generic patient characteristics, such as age [20] and sex [21], disease severity indicators, including the SOFA score [22], lactate level on first ICU admission [23], experience of septic shock [24] or ventilation [25], and complications associated with mortality in sepsis from the Cox regression analysis, were selected into the propensity score matching model, which was processed using fuzzy matching to create a 1 : 1 matching with 0.0028 toleration.

The blood iCa stage was defined according to the clinical regulations of our department: normal iCa range (1.15∼1.30 mmol/L), mild hypocalcemia (1.10∼1.15 mmol/L), moderate hypocalcemia (1.00∼1.10 mmol/L), and severe hypocalcemia (<1.00 mmol/L), and hypercalcemia (>1.30 mmol/L). The length of stay (LOS) in the ICU and hospital between the calcium-supplemented and nonsupplemented groups was compared using multiple comparisons. To investigate which particular situations were suitable for calcium supplementation, each interval in the stratified analysis was evaluated according to the intersections of the mortality curves of the two matching groups. The SOFA scores were categorized into the following intervals: ≤4, 5∼7, and ≥8. iCa was categorized into the following intervals: ≤1.00, 1.01∼1.20, and >1.20 mmol/L. The survival analysis for the calcium-supplemented and nonsupplemented cohorts in each interval was performed using landmark survival analysis to determine the effect of calcium supplementation on hospital mortality. The Kaplan–Meier survival curves are depicted.

Statistical analyses were performed using IBM SPSS software (version 25). Two-tailed *p* < 0.05 indicated a statistically significant difference.

## 3. Results

### 3.1. iCa at First ICU Admission Correlated with Mortality in Septic Patients

A total of 5,761 ICU admissions met our inclusion criteria and were included in the analysis. A total of 2,694 patients had the iCa data from their first ICU admission: 1,889 (70.12%) patients had hypocalcemia, 716 (26.58%) patients had a normal iCa range, and only 89 (3.30%) patients had hypercalcemia (Table 1). There were 2,194 survivors and 500 nonsurvivors (mortality: 18.56%) (Table 2). iCa at the first ICU admission was significantly lower in nonsurvivors than in survivors (1.09 (1.00∼1.14) vs. 1.10 (1.04∼1.15), *p*=0.006). There were more nonsurvivors than survivors with congestive heart failure (26.20% vs. 21.19%, *p*=0.015), cardiac arrhythmia (46.40% vs. 33.59%, *p* < 0.001), renal failure (20.00% vs. 15.22%, *p*=0.009), liver disease (30.20% vs. 13.31%, *p* < 0.001), metastatic cancer (10.00% vs. 5.29%, *p* < 0.001), coagulopathy (28.40% vs. 15.77%, *p* < 0.001), and fluid electrolyte disturbance (65.60% vs. 43.30%, *p* < 0.001). Variables with *p* < 0.1 were entered into the Cox regression model, and the results showed that iCa at the first ICU admission (HR: 0.421; 95% CI: 0.211∼0.837), age (HR: 1.027; 95% CI: 1.020∼1.033), cardiac arrhythmias (HR: 1.286; 95% CI: 1.059∼1.562), metastatic cancer (HR: 1.961; 95% CI: 1.455∼2.641), liver disease (HR: 2.059; 95% CI: 1.665∼2.544), and fluid electrolyte levels (HR: 1.453; 95% CI: 1.197∼1.765) were significantly associated with hospital mortality in septic patients (Table 3). These results suggest that hypocalcemia is common in sepsis, and the decrease in iCa at the first ICU admission poses an increased risk of mortality.

### 3.2. Longer Hospitalization in Septic Patients with Calcium Supplementation

**Figure 2:**
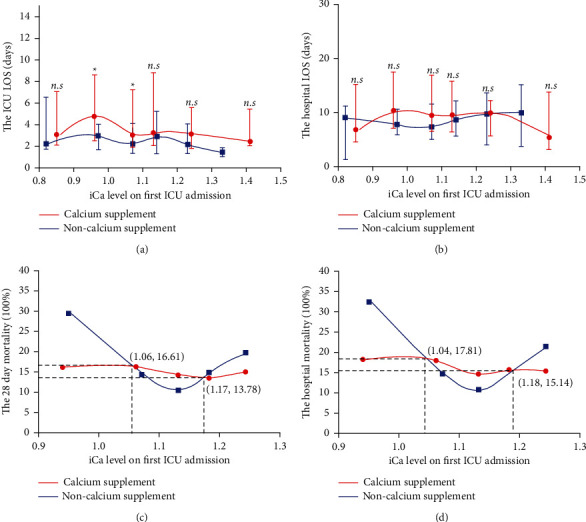
LOS and mortality in septic patients with or without calcium supplementation stratified by iCa on the first day of ICU admission. The LOS in the ICU (a) and hospital (b) in the calcium supplement group was longer than that in the nonsupplemented group. The data for each interval are expressed as the median with the interquartile range and connected using the LOWESS smoothing technique (Mann–Whitney *U* test, ^*∗*^*p* < 0.05; ^*∗∗*^*p* < 0.01; ^*∗∗∗*^*p* < 0.001; ^*∗∗∗∗*^*p* < 0.0001, and n.s. not significant); higher 28-day (c) and hospital (d) mortality were found in the calcium supplement group when iCa was within 1.04∼1.17 mmol/L, and lower 28-day (c) and hospital (d) mortality were found in the calcium supplement group when iCa was <1.04 or >1.17. The data for each interval are expressed as percentages and connected using the LOWESS smoothing technique.

**Figure 3:**
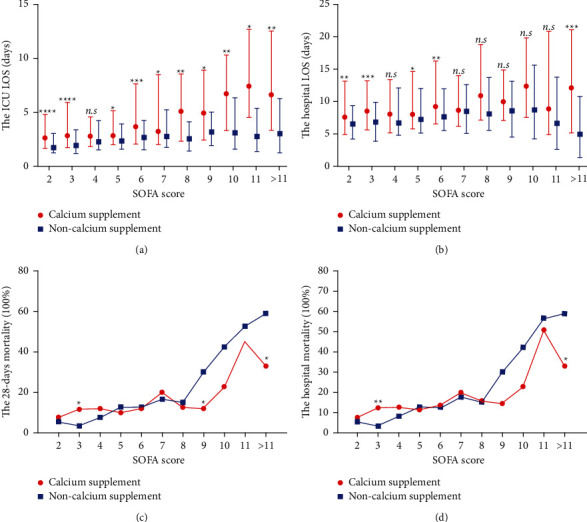
LOS and mortality in septic patients with or without calcium supplementation stratified by the SOFA score. The LOS in the ICU (a) and hospital (b) in the calcium-supplemented group was higher than that in the nonsupplemented group. The data for each interval are expressed as the median with the interquartile range (Mann–Whitney *U* test, ^*∗*^*p* < 0.05, ^*∗∗*^*p* < 0.01, ^*∗∗∗*^*p* < 0.001, ^*∗∗∗∗*^*p* < 0.0001, and n.s. not significant). A higher 28-day (c) and hospital (d) mortality were found in the calcium supplement group when the patient's SOFA score was ≥8, and a lower 28-day (c) and hospital (d) mortality were found in the calcium supplement group when the patient's SOFA score was ≤4. The data for each interval are expressed as percentages (chi-squared test, ^*∗*^*p* < 0.05, and ^*∗∗*^*p* < 0.01).

There were 2,689 (46.68%) patients who were admitted and received calcium supplementation and 3,072 (53.32%) patients who were admitted and did not receive calcium supplementation during the ICU stay. After propensity score matching according to age, sex, the SOFA score, lactate at the first ICU admission, and other complications associated with mortality in sepsis patients, 993 pairs of patients were ultimately included in the analysis. Overall, the ICU LOS was significantly longer in the calcium-supplemented group than in the nonsupplemented group (3.04 (1.83∼6.93) vs. 2.11 (1.24∼3.95), *p* < 0.001), and similar results were observed for the hospital LOS (8.26 (5.37∼14.11) vs. 6.87 (4.11∼10.88), *p* < 0.001) (Table 4). We stratified patients according to the SOFA score and iCa at the first ICU admission to examine whether the effect of calcium supplementation on hospitalization varied according to disease severity. The results showed that regardless of the SOFA score and iCa, most ICU (Figure 2(a) and Figure 3(a)) and hospital (Figures 2(b) and 3(b)) stays were longer in the calcium-supplemented group than in the nonsupplemented group, but some intervals were not significantly different between the matched groups.

### 3.3. Calcium Supplementation Exerts Positive and Negative Effects on the Mortality of Septic Patients

**Figure 4:**
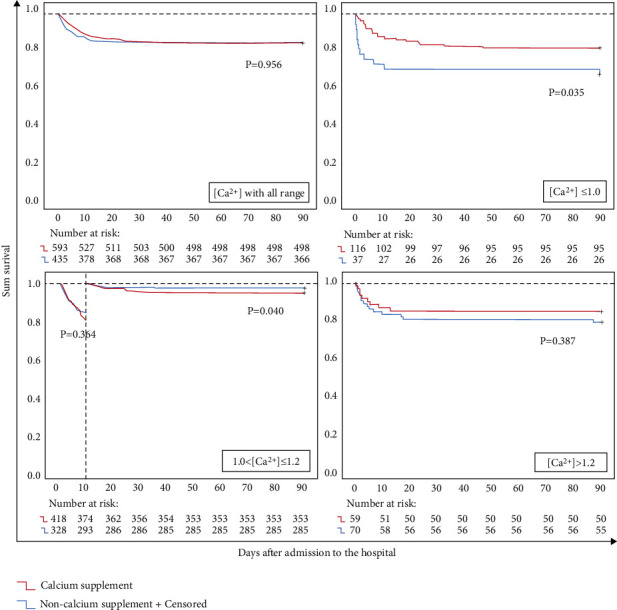
Landmark survival analysis of the difference in the 90-day mortality between the calcium-supplemented and nonsupplemented septic patients stratified by iCa at the first ICU admission.

We compared the mortality curves between the calcium supplement group and the nonsupplement group. The mortality of the matching groups in each iCa interval is shown in Table 5. Overall, the 28-day and hospital mortality in the calcium-supplemented group were higher than those in the nonsupplemented group (17.03% vs. 15.96% and 18.32% vs. 16.25%, respectively). The mortality curve for the 28-day (Figure 2(c)) and hospital (Figure 2(d)) patients showed a *U*-shaped curve, which suggests that an iCa level that is too high or too low increases the mortality risk for septic patients. However, the minimum of this curve was located in the mild hypocalcemia interval and not in the normal iCa range, and administration of calcium supplementation during this interval would increase mortality. This hypothesis was confirmed in the landmark analysis, which showed that administration of calcium supplementation to patients with an iCa level within 1.01∼1.20 mmol/L yielded a lower survival rate at the later stage of disease (*p*=0.040) (Figure 4). These results suggest that mild hypocalcemia is protective. In contrast, a lower mortality rate was observed when iCa was <1.01 or >1.20 mmol/L.

**Figure 5:**
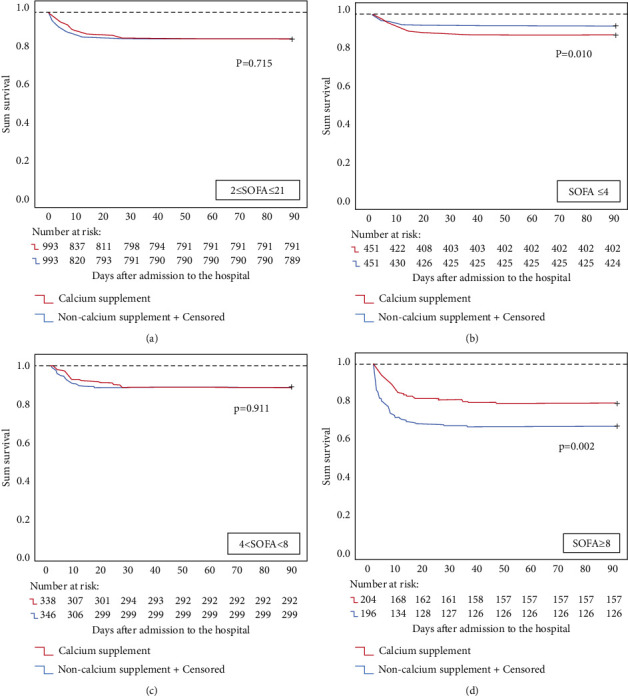
Landmark survival analysis of the difference in the 90-day mortality between calcium-supplemented and nonsupplemented septic patients stratified by the SOFA score.

The 28-day (Figure 3(c)) and hospital (Figure 3(d)) mortality progressively increased with increasing SOFA scores in the matching groups that were stratified by the SOFA score. The 28-day and hospital mortality rates between the matching groups were not significantly different after propensity score matching (13.49% vs. 14.30%, *p*=0.818, 14.5% vs. 14.6%, *p*=0.949, respectively) (Table 4). However, the results of the survival analysis showed that calcium supplementation reduced mortality when the patient's SOFA score was ≥8 (*p*=0.002), and higher mortality was observed with calcium supplementation when the SOFA score was ≤4 (*p*=0.010). There was no significant difference when the SOFA score was within the range of 5∼7 (*p*=0.911) (Figure 5), which suggested that calcium supplementation has the opposite effect on mortality in patients with different disease severities.

## 4. Discussion

Treatment of hypocalcemia in septic patients remains controversial. The international guidelines developed by the Surviving Sepsis Campaign provided no recommendations for calcium administration as a therapeutic measure [26]. Evidence-based guidance is lacking because no randomized controlled trials have investigated whether calcium supplementation should be administered to septic patients [13]. Unlike previous studies, our investigation assessed the prognosis of septic patients with or without calcium supplementation using stratified analyses of iCa and SOFA scores. We found that calcium supplementation exerted positive and negative effects on mortality for different SOFA score intervals, which may be clinically valuable because the SOFA score could be a substitute for iCa and could help clinicians make decisions regarding calcium supplementation when iCa measurements are unavailable.

The mortality curve in the nonsupplemented patients stratified by the iCa interval showed a *U*-shaped curve, which suggests that abnormal iCa poses a risk to septic patients. Because hypocalcemia is more prevalent in septic patients than hypercalcemia (in our case, 70.12% vs. 3.30%) and patients with severe hypocalcemia require critical care for a longer period [2], hypocalcemia may be another prognostic marker of sepsis. However, we found that the minimum point of this mortality curve was located at the mild hypocalcemia interval and not in the normal iCa range, which suggests that patients maintained under mild hypocalcemia may receive a benefit in terms of outcomes. Our recent experiment using a septic model also showed that mice pretreated with EDTA-2Na before cecal ligation and puncture (CLP) had 20∼50% lower mortality than those in the non-EDTA-2Na treatment group (unpublished data). Despite these findings, whether changes in blood iCa are a protective mechanism or simply a consequence of metabolic dysregulation when the body undergoes a critical illness must be established. If it is protective, then calcium supplementation may increase the burden on the body, which would downregulate iCa and worsen the outcome.

Overall, the ICU and hospital LOS in septic patients with calcium supplementation were longer than those in patients who did not receive supplementation. To examine whether the LOS varied across different subranges of iCa and disease severities, we stratified the LOS according to iCa and SOFA scores. However, the LOS tended to be higher in the calcium treatment groups for most subranges, particularly the ICU LOS, which suggests that calcium supplementation contributes to longer hospital stays. A similar phenomenon was seen in critically ill patients receiving calcium supplementation [14]. However, patients who stayed longer in the ICU may have a greater chance of receiving calcium supplementation and a higher likelihood of experiencing hypocalcemia. We could not exclude this possibility because causality could not be determined in our case. A large randomized controlled trial exploring the use of calcium supplementation in septic or ICU patients with different degrees of hypocalcemia is required to exclude this bias.

Unlike hospitalization, the two mortality curves crossed, which suggest that calcium supplementation exhibits the opposite effect on mortality on a case-by-case basis. It would be valuable for clinical decision-making to determine which situation is suitable for calcium supplementation. Therefore, the intersection of the mortality curves was calculated, and we found that patients receiving calcium supplementation tended to have higher hospital mortality when their iCa level at the first ICU admission was approximately 1.01∼1.20 mmol/L, which suggests that the attempted correction of blood calcium at this interval may be harmful. Most iCa levels at the first ICU admission for septic patients were located within this iCa interval, which may explain why some studies revealed that calcium supplementation had an adverse effect on the prognosis of septic patients [16, 27] and in septic models [17, 28]. However, there was a trend showing that calcium supplementation decreased mortality when iCa at the first ICU admission was <1.01 mmol/L or >1.20 mmol/L. However, we could not confirm this finding in the survival analysis, which may be due to the small number of patients in these subgroups.

The double effect of calcium supplementation was significant when the patients were stratified according to the SOFA score. Because iCa is fast-changing and not routinely measured in clinical practice, using the SOFA score as a substitute reference index for clinical calcium supplement decision-making may be valuable, particularly when iCa is not available. The incidence of severe hypocalcemia tends to be more pervasive in severe septic cases, and a negative correlation between the iCa concentration and mortality was observed in our study and previous studies [9, 10]. Moritoki et al. reported that the iCa concentration was not independently associated with the mortality, and only extreme iCa abnormalities were independent predictors of mortality [10]. Consistent with these results, calcium supplementation may benefit the outcome when the SOFA score is ≥8 and when patients experience severe hypocalcemia.

The underlying mechanism of the positive and negative effects of calcium supplementation in septic patients has not been well established. The administration of calcium supplementation to patients with severe hypocalcemia may prevent life-threatening complications, such as cardiac arrest or seizures, which may explain why patients with iCa levels <1.00 mmol/L had the highest mortality compared to patients with other iCa ranges. iCa also plays an important role in maintaining hemodynamics [29], and a direct relationship between iCa and arterial pressure was found in critically ill patients [30]. Calcium supplementation may improve hemodynamics by increasing the mean arterial pressure (MAP), the left ventricular stroke work index, and CO in critically ill patients [31, 32] and may improve heart function [33]. However, calcium supplementation may be deleterious at the cellular level because iCa may be shuttled into cells [34–36]. Therefore, the administration of additional parenteral calcium may aggregate the accumulation of cytosolic iCa concentrations, which would overactivate certain pathways and generate reactive oxygen species that trigger cell death [37]. Studies using calcium blockers as a treatment for sepsis showed an improvement in patient outcome [38–40], which supports this hypothesis. Therefore, the advantages and disadvantages should be weighed before calcium supplementation is administered to the patient. Notably, our study showed that calcium supplementation for patients with an iCa level at the first ICU admission >1.20 mmol/L tended to decrease mortality because excessive iCa levels are also a risk factor for increased mortality in septic patients. Notably, although hypocalcemia is very common in these patients, some do not present with hypocalcemia despite relatively high SOFA scores. These patients may not have an iCa influx problem. Therefore, it would be valuable to examine the contribution of calcium supplementation to hemodynamics in these individuals.

There are several limitations in this study. First, it was retrospective and purely observational. Some patients who may have received calcium supplementation or treatments such as fast transfusion that would affect blood iCa levels prior to admission to the ICU were not included in the analysis because the MIMIC-III is an ICU database. Second, the formula, dose, timing, and duration of calcium administration are important factors that influence the therapeutic effect of calcium replacement. Our approach did not investigate these factors because there were 5 types of calcium replacement agents in use in the MIMIC-III database, and hypocalcemia may have occurred at any time during their ICU stay, which led to the variation in the administration, time of administration and duration of administration from case to case. Therefore, our study only preliminarily analyzed whether calcium supplementation should be administered and under which conditions it is suitable for ICU septic patients. Third, an immortal time bias exists because patients who stayed longer in the ICU had a greater chance of experiencing hypocalcemia and receiving calcium supplementation. Although a longer hospitalization was noted in the calcium supplementation group, the causality between longer hospitalization and calcium supplementation could not be determined in our study. Fourth, calcium agents can be used for hypocalcemia therapy and other purposes and we did not identify the calcium agent used for hypocalcemia therapy or other purposes in the MIMIC-III database. Fifth, the dynamics of iCa may be fast-changing and hypocalcemia and hypercalcemia may occur in the same patient during their ICU stay [10]. Our study focused on iCa at the first ICU admission, which may be inadequate. However, we found that it was significantly correlated with mortality in septic patients. Finally, our study presented only a rough reference range of iCa and SOFA scores for calcium supplementation, and the sample size of patients with iCa <1.01 or >1.20 mmol/L was small. Therefore, the study was underpowered to produce convincing conclusions for this subgroup. Larger amounts of data are needed, and controlled intervention studies should be performed to assess the range in which this intervention should be applied for clinical decision-making.

## 5. Conclusions

Septic patients are at particular risk for hypocalcemia, and the effect of calcium administration on septic patients with different disease severities is largely unknown. We extracted the data from septic patients admitted to the ICU from the MIMIC-III database and compared the prognosis between calcium-supplemented and nonsupplemented patients. Our findings suggest that mild hypocalcemia is protective in septic patients. There were positive and negative effects of calcium supplementation on mortality in septic patients with different SOFA score intervals. Therefore, the SOFA score may be a valuable clinical index for decisions regarding calcium administration, particularly when iCa is not available. Further controlled intervention studies should be performed to verify the advantages and disadvantages of this intervention and apply them to clinical decision-making.

## Figures and Tables

**Table 1:** The clinical characteristics of septic patients with different levels of iCa at the first ICU admission.

Clinical parameters	Total (*n* = 2,694)	Hypocalcemia (<1.15 mmol/L; *n* = 1,889, 70.12%)	Normal (1.15∼1.30 mmol/L; *n* = 716, 26.58%)	Hypercalcemia (>1.30 mmol/L; *n* = 89, 3.30%)	*P* value
Age (years; median, *Q*_1_∼*Q*_3_)	64.40 (52.05∼77.19)	63.39 (50.82∼76.56)	66.92 (56.45∼78.30)	64.31 (53.87∼80.51)	<0.0001
Sex (male; *n*, %)	1579 (58.61)	1099 (58.18)	427 (59.64)	53 (59.55)	0.783
Ethnicity (*n*, %)					
White	1905 (70.71)	1312 (69.45)	534 (74.58)	59 (66.29)	0.024
Black	197 (7.31)	131 (6.93)	56 (7.82)	10 (11.24)	0.253
Hispanic/Latino	79 (2.93)	67 (3.54)	9 (1.26)	3 (3.37)	0.010
Others	513 (19.04)	379 (20.06)	117 (16.34)	17 (19.10)	0.097
Severity					
SOFA (mean ± SD)	6.33 ± 3.66	6.47 ± 3.80	5.84 ± 3.19	7.23 ± 3.87	0.001
SIRS (mean ± SD)	3.09 ± 0.87	3.13 ± 0.87	3.00 ± 0.86	3.00 ± 1.03	0.070
LODS (mean ± SD)	5.66 ± 3.16	5.71 ± 3.25	5.41 ± 2.86	6.62 ± 3.47	0.020
qSOFA (mean ± SD)	1.98 ± 0.67	1.96 ± 0.67	2.00 ± 0.68	2.05 ± 0.67	0.370
Septic shock (*n*, %)	423 (15.70)	339 (17.94)	74 (10.34)	10 (11.24)	<0.0001
Vent (*n*, %)	1998 (74.16)	1388 (73.47)	542 (75.70)	68 (76.64)	0.455
Lactate (median, *Q*_1_∼*Q*_3_)	1.90 (1.20∼3.10)	1.90 (1.30∼3.22)	1.70 (1.10∼2.60)	3.10 (1.80∼5.40)	<0.0001
28-day mortality (*n*, %)	478 (17.74)	354 (18.74)	104 (14.53)	20 (22.47)	0.008
Hospital mortality (*n*, %)	500 (18.53)	371 (19.64)	109 (15.22)	20 (22.47)	0.006
ICU LOS (days; median, *Q*_1_∼*Q*_3_)	3.37 (1.86∼7.27)	3.57 (1.90∼7.79)	3.10 (1.66∼6.23)	2.84 (1.761∼5.68)	0.002
Hospital LOS (days; median, *Q*_1_∼*Q*_3_)	8.98 (5.44∼15.01)	8.97 (5.36∼15.44)	8.95 (5.71∼13.87)	9.69 (5.16∼13.66)	0.861

SOFA, sequential organ failure assessment; qSOFA, quick sequential organ failure assessment; SIRS, systemic inflammatory response syndrome, LODS, logistic organ dysfunction system; ICU LOS, intensive care unit length of stay; Hospital LOS, hospital length of stay.

**Table 2:** Differences in clinical characteristics between survivors and nonsurvivors in septic patients with iCa records at the first ICU admission (hospital mortality).

	Total (*n* = 2,694)	Survivors (*n* = 2,194)	Nonsurvivors (*n* = 500)	*P* value
Age (median, *Q*_1_∼*Q*_3_)	64.46 (52.05–77.19)	63.38 (51.17–75.25)	70.3 6 (57.23–81.73)	<0.001
Sex (male; *n*, %)	1579 (58.61%)	1291 (58.84%)	288 (57.60%)	0.611
Ca^2+^ on first ICU admission (median, *Q*_1_∼*Q*_3_)	1.10 (1.04∼1.15)	1.10 (1.04∼1.15)	1.09 (1.00∼1.14)	0.006
Congestive heart failure (*n*, %)	596 (22.12%)	465 (21.19%)	131 (26.20%)	0.015
Cardiac arrhythmias (*n*, %)	979 (36.34%)	737 (33.59%)	242 (48.40%)	<0.001
Pulmonary circulation (*n*, %)	212 (7.87%)	174 (7.93%)	38 (7.60%)	0.804
Hypertension (*n*, %)	1532 (56.87%)	1255 (57.20%)	277 (55.40%)	0.463
Chronic pulmonary (*n*, %)	604 (22.42%)	482 (21.97%)	122 (24.40%)	0.240
Diabetes uncomplicated (*n*, %)	565 (20.97%)	459 (20.92%)	106 (21.20%)	0.890
Diabetes complicated (*n*, 3%)	156 (5.79%)	126 (5.74%)	30 (6.00%)	0.824
Hypothyroidism (*n*, %)	328 (12.18%)	261 (11.90%)	67 (13.40%)	0.353
Renal failure (*n*, %)	434 (16.11%)	334 (15.22%)	100 (20.00%)	0.009
Liver disease (*n*, %)	443 (16.44%)	292 (13.31%)	151 (30.20%)	<0.001
Metastatic cancer (*n*, %)	166 (6.16%)	116 (5.29%)	50 (10%)	<0.001
Coagulopathy (*n*, %)	488 (18.11%)	346 (15.77%)	142 (28.40%)	<0.001
Fluid electrolyte disturbance (*n*, %)	1278 (47.44%)	950 (43.30%)	328 (65.60%)	<0.001

**Table 3:** Cox regression analysis showing variables associated with hospital mortality.

	B	S.E.	Wald	*P* value	HR	95% CI for HR	
						Lower	Upper
Age	0.026	0.003	62.956	<0.001	1.027	1.020	1.033
iCa at first ICU admission	−0.866	0.351	6.075	0.014	0.421	0.211	0.837
Congestive heart failure	0.100	0.110	0.833	0.361	1.105	0.891	1.371
Cardiac arrhythmias	0.252	0.099	6.444	0.011	1.286	1.059	1.562
Renal failure	0.082	0.117	0.497	0.481	1.086	0.863	1.366
Liver disease	0.722	0.108	44.603	<0.001	2.059	1.665	2.544
Coagulopathy	0.159	0.108	2.170	0.141	1.172	0.949	1.448
Metastatic cancer	0.673	0.152	19.607	<0.001	1.961	1.455	2.641
Fluid electrolyte	0.374	0.099	14.209	<0.001	1.453	1.197	1.765

**Table 4:** The clinical characteristics of septic patients with or without calcium supplementation (before and after propensity score matching).

Clinical parameters	Before propensity score matching			After propensity score matching		
	Calcium supplementation (*n* = 2,689)	Nonsupplementation (*n* = 3,072)	*P* value	Calcium supplementation (*n* = 993)	Nonsupplementation (*n* = 993)	*P* value
Age (years; median, *Q*1∼*Q*3)	64.85 (52.01∼77.82)	68.77 (55.95∼81.51)	<0.001	66.91 (53.49∼79.96)	67.14 (55.56∼79.58)	0.553
Sex (male, %)	1541 (57.31)	1672 (54.43)	0.013	541 (54.48%)	529 (53.27%)	0.589

Complications (*n*, %)						
Cardiac arrhythmias	988 (36.74%)	1124 (36.6%)	0.911	347 (34.94%)	364 (36.66%)	0.426
Liver disease	470 (17.48%)	437 (14.23%)	0.001	152 (15.31%)	154 (15.51%)	0.901
Renal failure	491 (18.26%)	647 (21.07%)	0.008	187 (18.83%)	194 (19.54%)	0.690
Fluid electrolyte	1392 (51.77%)	1304 (42.46%)	<0.001	497 (50.05%)	480 (48.34%)	0.445
Metastatic cancer	166 (6.17%)	229 (7.46%)	0.054	76 (7.65%)	81 (8.16%)	0.678

Severity						
Lactate at the first hospital admission (median, *Q*1∼*Q*3)	1.90 (1.30∼3.10)	1.70 (1.2∼2.5)	<0.001	1.8 (1.2∼2.6)	1.8 (1.2∼2.6)	0.474
Septic_shock_explicit (*n*, %)	472 (17.55%)	266 (8.66%)	<0.001	142 (14.3%)	135 (13.6%)	0.650
Vent (*n*, %)	1659 (61.7%)	1120 (36.46%)	<0.001	466 (46.93%)	477 (48.04%)	0.621
SOFA (mean ± SD)	6.05 ± 3.611	4.85 ± 2.71	<0.001	5.40 ± 2.92	5.40 ± 2.90	0.939
28-day mortality (*n*, %)	442 (16.43)	352 (11.46%)	0.005	134 (13.49%)	142 (14.30%)	0.818
Hospital mortality (*n*, %)	471 (17.52)	361 (11.75%)	<0.001	144 (14.5%)	145 (14.6%)	0.949
ICU LOS (days; median, *Q*1∼*Q*3)	3.65 (1.87∼7.97)	2.01 (1.12∼3.64)	<0.001	3.04 (1.83∼6.93)	2.11 (1.24∼3.95)	<0.001
Hospital LOS (days; median, *Q*1∼*Q*3)	8.99 (5.54∼15.67)	6.57 (3.84∼10.59)	<0.001	8.26 (5.37∼14.11)	6.87 (4.11∼10.88)	<0.001

SOFA, sequential organ failure assessment; qSOFA, quick sequential organ failure assessment; ICU LOS, intensive care unit length of stay; hospital LOS, hospital length of stay.

**Table 5:** The difference in the 28-day and hospital mortality in septic patients with or without calcium supplementation under different iCa intervals.

iCa range (mmol/L)	Calcium supplementation (*n* = 593)			Nonsupplementation (*n* = 435)		
	iCa at the first ICU admission (median, *Q*_1_∼*Q*_3_)	28-day mortality (%)	Hospital mortality (%)	iCa at the first ICU admission (median, *Q*_1_∼*Q*_3_)	28-day mortality (%)	Hospital mortality (%)
≤1.00	0.94 (0.89∼0.98)	16.37	18.10	0.95 (0.90∼0.97)	29.72	32.43
1.01∼1.10	1.06 (1.04∼1.09)	16.52	17.80	1.07 (1.04∼1.09)	14.56	14.56
1.11∼1.15	1.13 (1.12∼1.14)	14.52	14.52	1.13 (1.12∼1.14)	10.69	10.69
1.16–1.20	1.18 (1.17∼1.19)	13.79	15.52	1.18 (1.17∼1.20)	15.15	15.15
≥1.20	1.24 (1.23∼1.27)	15.25	15.25	1.24 (1.22∼1.30)	20.00	21.43
Total	1.09 (1.02∼1.14)	15.18	16.36	1.12 (1.06∼1.18)	15.40	16.09

## Data Availability

The calcium supplementation and noncalcium supplementation patient datasets in this study are available after requirement since the Excel files cannot be uploaded in this submission platform.

## Supplementary Materials

Supplementary Materials The details on the ROC curve were uploaded as the Word file named Supplement table 1. The corresponding weights of the component variables of the PS model were uploaded as the Word file named Supplement table 2. The unmatched analysis files named Supplement Figures 2–5 were also added to the Supplementary material.
