# Supplementary material for: The Positive and Negative Effects of Calcium Supplementation on Mortality in Septic ICU Patients Depend on Disease Severity: A Retrospective Study from the MIMIC-III
Source: Crit Care Res Pract. 2022 Jun 22;2022:2520695. doi: 10.1155/2022/2520695 (PMC9242801; doi:10.1155/2022/2520695)

Supplement table 1. The ROC of the covariates and its conjoint factor in PS model

| Test Result Variable(s) | AUC | Std. Error | *P* | 95% CI | |
| --- | --- | --- | --- | --- | --- |
|  |  |  |  | Lower Bound | Upper Bound |
| Age | 0.617 | 0.015 | <0.001 | 0.587 | 0.647 |
| Sex | 0.493 | 0.016 | 0.667 | 0.463 | 0.524 |
| Sofa score | 0.745 | 0.014 | <0.001 | 0.718 | 0.772 |
| Lactate on first ICU admission | 0.692 | 0.015 | <0.001 | 0.663 | 0.722 |
| Septic shock | 0.608 | 0.016 | <0.001 | 0.576 | 0.640 |
| Ventilation | 0.571 | 0.015 | <0.001 | 0.542 | 0.600 |
| Cardiac arrhythmias | 0.582 | 0.016 | <0.001 | 0.552 | 0.613 |
| Liver disease | 0.590 | 0.016 | <0.001 | 0.558 | 0.621 |
| Renal failure | 0.513 | 0..011 | 0.227 | 0.492 | 0.535 |
| Fluid electrolyte | 0.608 | 0.015 | <0.001 | 0.579 | 0.638 |
| Metastatic cancer | 0.524 | 0.016 | 0.125 | 0.493 | 0.555 |
| Conjoint factor | 0.823 | 0.011 | <0.001 | 0.801 | 0.845 |


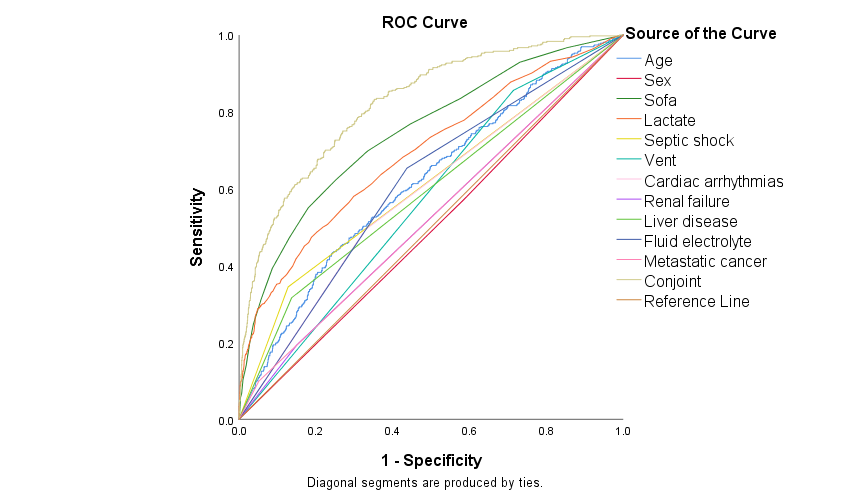

Supplement: Supplementary Materials — The details on the ROC curve were uploaded as the Word file named Supplement table 1. The corresponding weights of the component variables of the PS model were uploaded as the Word file named Supplement table 2. The unmatched analysis files named Supplement Figures 2–5 were also added to the Supplementary material. [file 2520695.f1.zip › 2520695.f1/Supplement table 1. The ROC of the covariates in PS model.docx]
