# Supplementary material for: The Positive and Negative Effects of Calcium Supplementation on Mortality in Septic ICU Patients Depend on Disease Severity: A Retrospective Study from the MIMIC-III
Source: Crit Care Res Pract. 2022 Jun 22;2022:2520695. doi: 10.1155/2022/2520695 (PMC9242801; doi:10.1155/2022/2520695)
Supplement: Supplementary Materials — The details on the ROC curve were uploaded as the Word file named Supplement table 1. The corresponding weights of the component variables of the PS model were uploaded as the Word file named Supplement table 2. The unmatched analysis files named Supplement Figures 2–5 were also added to the Supplementary material. [file 2520695.f1.zip › 2520695.f1/Supplement Table 2. The weight of the covariates.docx]

**Supplement Table 2**. The weight of the covariates in PS model (analyzed by using Linear regression (Stepwise))

| **Coefficients^a^** | | | | | | | |
| --- | --- | --- | --- | --- | --- | --- | --- |
| Model | Unstandardized Coefficients | | Standardized Coefficients | t | *P* | Collinearity Statistics | |
|  | B | Std. Error | Beta |  |  | Tolerance | VIF |
| (Constant) | -0.307 | 0.025 |  | -12.531 | <0.001 |  |  |
| Age | 0.003 | 0.000 | 0.131 | 8.052 | <0.001 | 0.839 | 1.192 |
| Sofa | 0.025 | 0.002 | 0.230 | 12.380 | <0.001 | 0.643 | 1.556 |
| Septic shock | 0.052 | 0.016 | 0.053 | 3.248 | 0.001 | 0.847 | 1.180 |
| Vent | 0.055 | 0.012 | 0.075 | 4.688 | <0.001 | 0.860 | 1.162 |
| Lactate | 0.033 | 0.003 | 0.181 | 11.195 | <0.001 | 0.852 | 1.174 |
| Metastatic cancer | 0.142 | 0.021 | 0.101 | 6.719 | <0.001 | 0.990 | 1.011 |
| Liver disease | 0.061 | 0.016 | 0.061 | 3.786 | <0.001 | 0.850 | 1.177 |
| Cardiac arrhythmias | 0.032 | 0.012 | 0.042 | 2.670 | 0.008 | 0.890 | 1.124 |

a. Dependent Variable: hospital mortality
