# Supplementary figures and images for: The Positive and Negative Effects of Calcium Supplementation on Mortality in Septic ICU Patients Depend on Disease Severity: A Retrospective Study from the MIMIC-III
Source: Crit Care Res Pract. 2022 Jun 22;2022:2520695. doi: 10.1155/2022/2520695 (PMC9242801; doi:10.1155/2022/2520695)

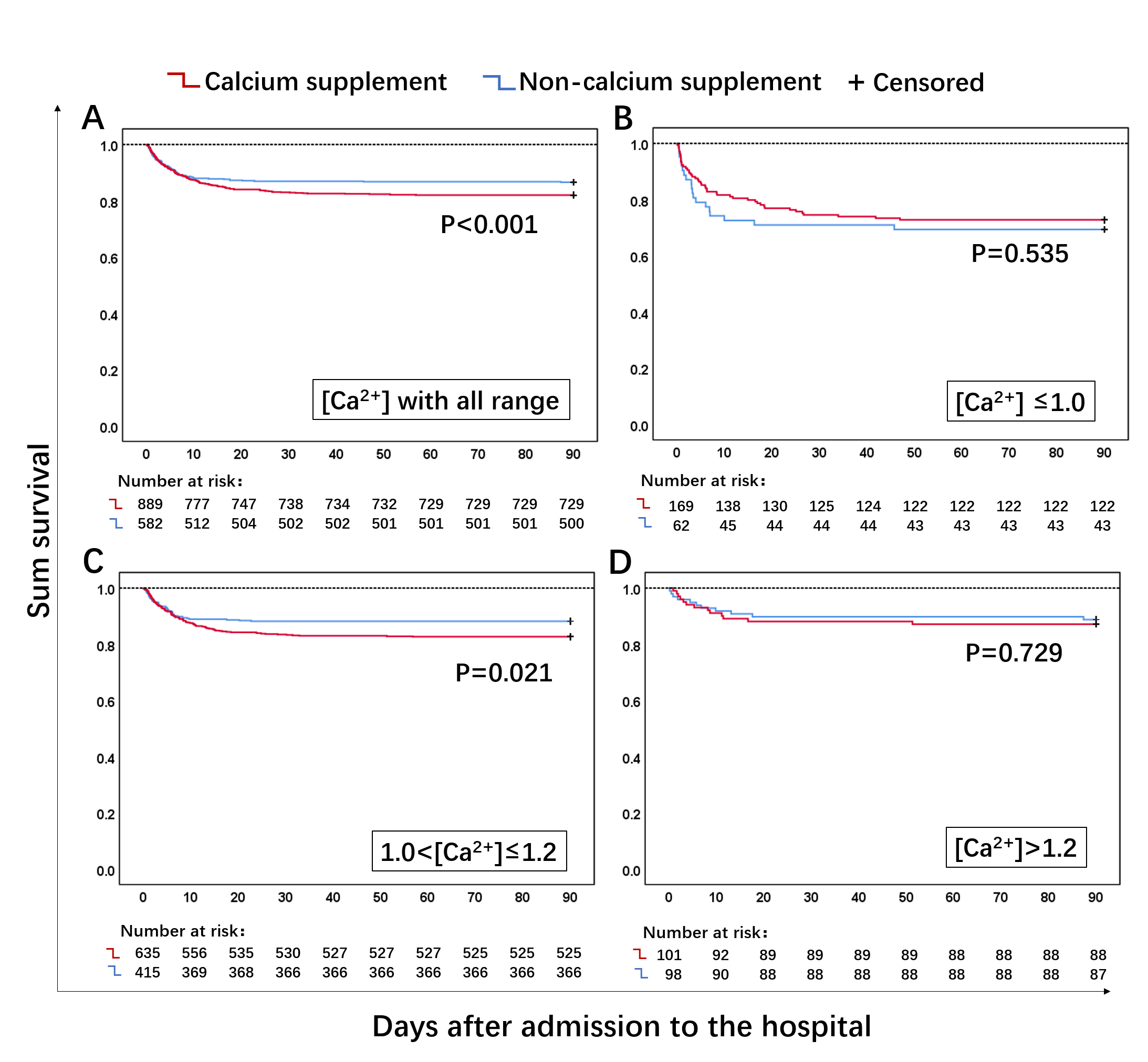

Supplement: Supplementary Materials — The details on the ROC curve were uploaded as the Word file named Supplement table 1. The corresponding weights of the component variables of the PS model were uploaded as the Word file named Supplement table 2. The unmatched analysis files named Supplement Figures 2–5 were also added to the Supplementary material. [file 2520695.f1.zip › 2520695.f1/Supplement Fig 3 (unmatched analysis) (1).png]

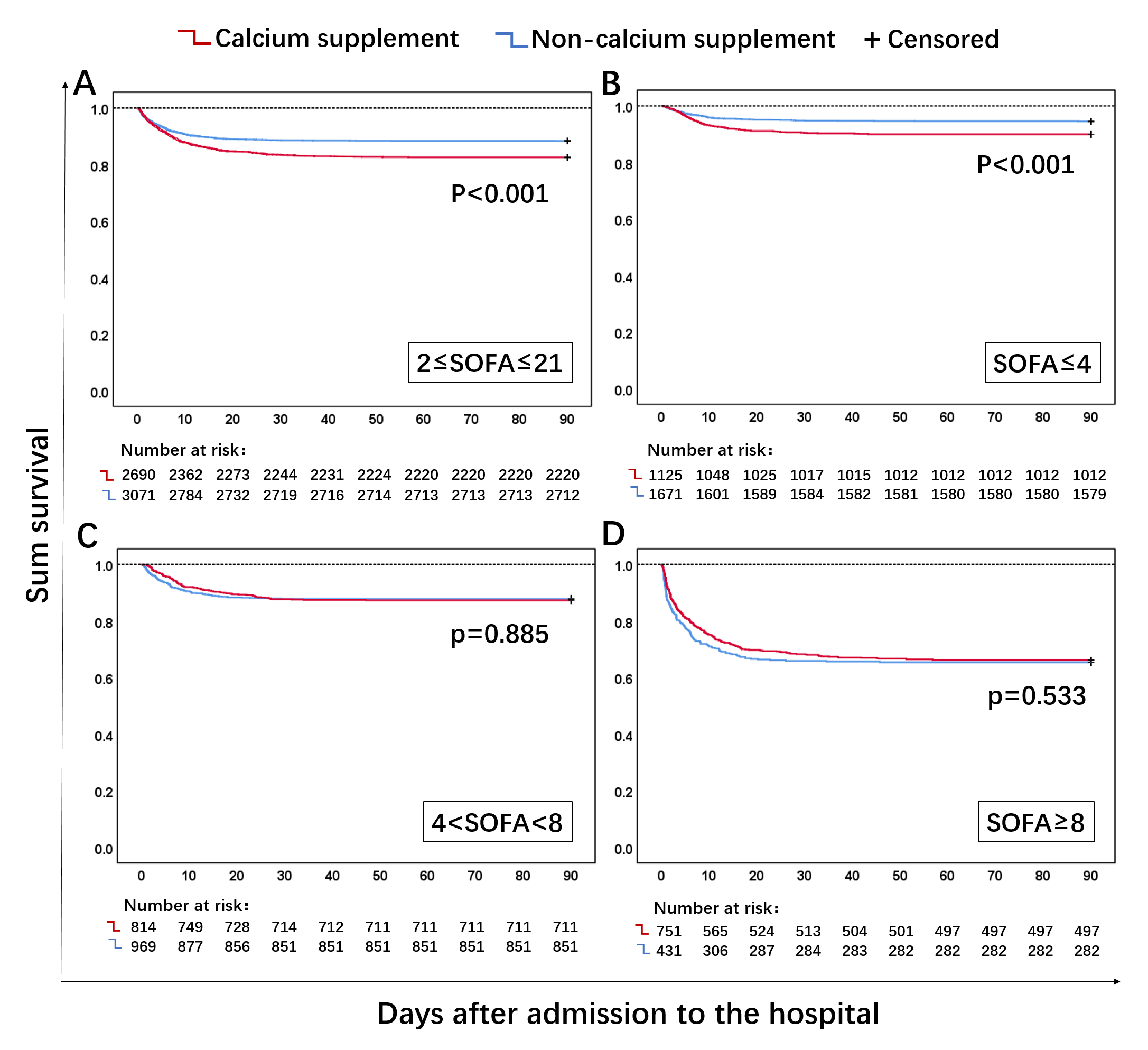

Supplement: Supplementary Materials — The details on the ROC curve were uploaded as the Word file named Supplement table 1. The corresponding weights of the component variables of the PS model were uploaded as the Word file named Supplement table 2. The unmatched analysis files named Supplement Figures 2–5 were also added to the Supplementary material. [file 2520695.f1.zip › 2520695.f1/Supplement Fig 5 (unmatched analysis) (1).png]
